# High spatiotemporal variability of methane concentrations challenges estimates of emissions across vegetated coastal ecosystems

**DOI:** 10.1111/gcb.16177

**Published:** 2022-04-12

**Authors:** Florian Roth, Xiaole Sun, Marc C. Geibel, John Prytherch, Volker Brüchert, Stefano Bonaglia, Elias Broman, Francisco Nascimento, Alf Norkko, Christoph Humborg

**Affiliations:** ^1^ 7675 Baltic Sea Centre Stockholm University Stockholm Sweden; ^2^ 3835 Tvärminne Zoological Station University of Helsinki Hanko Finland; ^3^ Center of Deep Sea Research Institute of Oceanology Chinese Academy of Sciences Qingdao China; ^4^ 7675 Department of Meteorology Stockholm University Stockholm Sweden; ^5^ 7675 Department of Geological Sciences Stockholm University Stockholm Sweden; ^6^ 7675 Bolin Centre for Climate Research Stockholm University Stockholm Sweden; ^7^ Department of Marine Sciences University of Gothenburg Gothenburg Sweden; ^8^ 7675 Department of Ecology Environment and Plant Sciences Stockholm University Stockholm Sweden

**Keywords:** blue carbon, carbon cycle, climate change, coastal greenhouse gas emissions, methane fluxes

## Abstract

Coastal methane (CH_4_) emissions dominate the global ocean CH_4_ budget and can offset the “blue carbon” storage capacity of vegetated coastal ecosystems. However, current estimates lack systematic, high‐resolution, and long‐term data from these intrinsically heterogeneous environments, making coastal budgets sensitive to statistical assumptions and uncertainties. Using continuous CH_4_ concentrations, δ^13^C‐CH_4_ values, and CH_4_ sea–air fluxes across four seasons in three globally pervasive coastal habitats, we show that the CH_4_ distribution is spatially patchy over meter‐scales and highly variable in time. Areas with mixed vegetation, macroalgae, and their surrounding sediments exhibited a spatiotemporal variability of surface water CH_4_ concentrations ranging two orders of magnitude (i.e., 6–460 nM CH_4_) with habitat‐specific seasonal and diurnal patterns. We observed (1) δ^13^C‐CH_4_ signatures that revealed habitat‐specific CH_4_ production and consumption pathways, (2) daily peak concentration events that could change >100% within hours across all habitats, and (3) a high thermal sensitivity of the CH_4_ distribution signified by apparent activation energies of ~1 eV that drove seasonal changes. Bootstrapping simulations show that scaling the CH_4_ distribution from few samples involves large errors, and that ~50 concentration samples per day are needed to resolve the scale and drivers of the natural variability and improve the certainty of flux calculations by up to 70%. Finally, we identify northern temperate coastal habitats with mixed vegetation and macroalgae as understudied but seasonally relevant atmospheric CH_4_ sources (i.e., releasing ≥ 100 μmol CH_4_ m^−2^ day^−1^ in summer). Due to the large spatial and temporal heterogeneity of coastal environments, high‐resolution measurements will improve the reliability of CH_4_ estimates and confine the habitat‐specific contribution to regional and global CH_4_ budgets.

## Graphical abstract

Shallow coastal waters are hotspots for ocean methane emissions, but the fluxes remain poorly constrained in space and time due to the heterogeneous and dynamic nature of these environments. Using continuous measurements, we identify unprecedented spatiotemporal variability in water‐column methane distribution that range orders of magnitude, with habitat‐specific production and consumption pathways, seasonal temperature dependencies, and short‐term fluctuations. We discuss how failing to include spatially and temporally resolved assessments in future studies will result in a continued systematic bias of emission estimates and propose sampling intensities that help determine the scale and drivers of this variability across the coastal seascape.

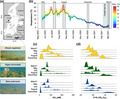

## 1 INTRODUCTION

Methane (CH_4_) is the second most important greenhouse gas (GHG) driving global climate change (Shindell et al., 2009). Past research has shown that coastal marine environments dominate the global ocean CH_4_ budget and contribute 5–28 Tg CH_4_ yr^−1^ to total global CH_4_ emissions (Rosentreter et al., 2021b; Weber et al., 2019). However, a scarcity of systematic, high‐resolution, and long‐term measurements has hampered our ability to confine CH_4_ emissions from a wide range of heterogeneous and dynamic coastal environments impeding efforts to evaluate the potential of coastal ecosystems to mitigate climate change by storing carbon (Rosentreter et al., 2021a).

Particularly in coastal sediments, CH_4_ can be produced in large amounts due to the organic carbon surplus of primary production from submerged (e.g., seagrass and macroalgae) and partially emerged (e.g., mangroves and salt marshes) vegetation (Duarte et al., 2005; Ortega et al., 2019; Snelgrove et al., 2018) and the accumulation of allochthonous particulate organic matter (Barnes & Goldberg, 1976; Reeburgh, 1983; Wallenius et al., 2021). In such environments, CH_4_ emissions can offset or even negate the value of coastal ecosystems as "blue carbon" storage reservoirs by counteracting carbon fixation and burial (Rosentreter et al., 2018). However, stretching over a global coastline of ~1,600,000 km, these environments are intrinsically heterogeneous with a mosaic of habitats with varying substrate composition (Holland & Elmore, 2008; Koch, 2001), associated species communities (Dias et al., 2018; Stein et al., 2014), and ecosystem processes across space and time (Cardinale et al., 2006; Hewitt et al., 2008). Thus, the inherent properties that make coastal environments so diverse and heterogenous also complicate our ability to narrow down carbon dynamics in these areas sufficiently (Rosentreter et al., 2021a).

In this context, global estimates of coastal CH_4_ emissions presently do not sufficiently reflect the heterogeneous and dynamic nature of coastal environments. In fact, the three classical blue carbon ecosystems, seagrass meadows, salt marshes, and mangrove forests (Mcleod et al., 2011) have been the focal point for global coastal CH_4_ assessments due to their large carbon sequestration potential (Mcleod et al., 2011). It is only recently that tidal flats, coastal aquaculture, and inner estuaries have been added to the global CH_4_ budget (Rosentreter et al., 2021b), but measurements from other coastal areas are still pooled without further habitat discrimination (Weber et al., 2019). For example, highly productive but less conspicuous coastal ecosystems with mixed‐macrophytes, algal dominance, or bare sediments are common but not explicitly included in these estimates. Yet, given their high carbon turnover rates (Attard et al., 2019a, 2019b), these habitats may contribute significantly to the total coastal CH_4_ emissions (Lundevall‐Zara et al., 2021). Moreover, the majority (85%) of studies quantifying CH_4_ emissions from vegetated coastal areas have been performed south of 45 degrees North (70% when excluding mangroves that only occur around the tropics) (Al‐Haj & Fulweiler, 2020). Northern temperate and high‐latitude coastal systems are highly productive and also experience climate change at an accelerated rate compared to low‐ and mid‐latitude areas (Screen & Simmonds, 2010; Serreze et al., 2009), increasing the demand for studies assessing temperature‐sensitive CH_4_ dynamics from these regions (Yvon‐Durocher et al., 2014). There is also a major knowledge gap in our understanding of the variability of CH_4_ in surface waters over short spatial scales reflecting the ecosystem mosaic typical for the coastal environment (Sheaves, 2009), as has been shown relevant for seafloor gross primary production and community respiration in shallow areas (Rodil et al., 2021).

Emissions of CH_4_ are, furthermore, particularly variable in time, and narrowing the uncertainty in the global coastal CH_4_ budget requires a methodology capable of quantifying natural variations arising from biotic and abiotic drivers across multiple timescales (Rosentreter et al., 2021a). For example, 74 of 98 studies (75.5%) used to calculate the global median CH_4_ flux from vegetated coastal ecosystems in Al‐Haj and Fulweiler (2020) employed flux chamber measurements or discrete sampling. Chamber measurements produce time‐averaged flux estimates (often for a period between 24 and 48 h). In contrast, discrete samples have no time‐weighted average, but due to logistical reasons, are usually taken at frequencies of no more than one to five samples per day and location (Banerjee et al., 2018; Dutta et al., 2015; Nirmal Rajkumar et al., 2008). These studies resulted in significant advances in our understanding of CH_4_ emission from coastal systems. Yet, the strong influence of physical forcing (e.g., wind, waves, currents, tides) on the main CH_4_ emission pathways (diffusion and ebullition) over short timescales (minutes to hours) can lead to a high CH_4_ concentration and flux variability within one diel cycle, as has been shown in lake environments (Sieczko et al., 2020) and tidal influenced estuarine systems (Rosentreter et al., 2018). In the past decade, methods have been developed to improve the spatial and temporal resolutions of CH_4_ concentration and flux measurements in aquatic systems. For example, using real‐time in situ measurements based on a gas equilibrator coupled to cavity ring‐down spectroscopy (CRDS), Call et al. (2015) and (2019) showed variability across days to weeks and Rosentreter et al. (2018) documented seasonal CH_4_ variability in mangrove creeks. These high‐resolution efforts have facilitated an improved understanding of different pathways, sources, and sinks in mangrove forests, yet the amplitude and underlying mechanisms of this variability in other coastal marine ecosystems are largely unknown. In addition, seasonal sampling becomes especially important for annual estimates from underrepresented northern temperate and high‐latitude regions, but time‐series measurements are often discontinued in winter due to harsh weather conditions.

Although high‐resolution measurements are critical for reliably capturing the magnitude of the coastal CH_4_ variability, these sampling campaigns can be time‐consuming and expensive. As such, it is desirable to determine the sampling effort required to obtain a high‐accuracy, representative mean dissolved CH_4_ concentration for various coastal environments.

**FIGURE 1:**
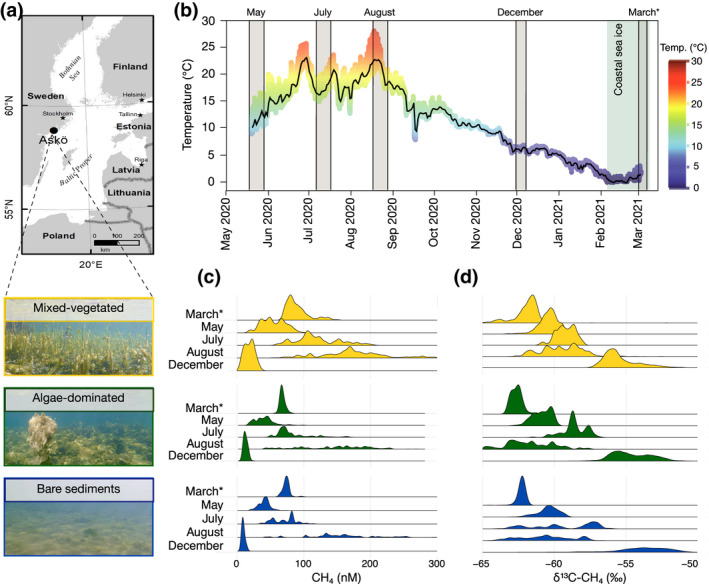
Study location and habitat types (a), surface water temperature at the study location (b) and histograms of the density distributions of surface water methane (CH_4_) concentrations (c) and stable carbon isotopes of CH_4_ (d) across habitats and different sampling months. The five sampling campaigns are depicted as grey bars in (b); the light blue bar indicates the period of coastal sea ice cover. Temperature‐coded points are individual measurements at 15 min intervals, and the black line denotes the daily running mean temperature. CH_4_ concentrations in (c) >300 nM, which represent <1% of the data, were omitted for graphical representation but can be found in Table 1. The asterisk denotes under‐ice sampling in March

We explored the spatial and temporal variability of CH_4_ across various heterogeneous coastal environments by systematically measuring CH_4_ concentrations in three widely distributed yet understudied northern temperate coastal habitats (Figure 1a). The CH_4_ distribution in shallow (<4 m water depth) mixed‐vegetated, algae‐dominated, and adjacent bare sediment habitats was assessed during five sampling campaigns spanning an entire year (Figure 1b), including an ice‐covered period in late winter/early spring. We performed in situ real‐time monitoring of CH_4_ concentrations using CRDS to account for the temporal variability by diel cycles and peak events (Call et al., 2015; Maher et al., 2013; Rosentreter et al., 2018). This state‐of‐the‐art technique also permits high temporal resolution measurements of stable carbon isotope ratios of CH_4_ (δ^13^C‐CH_4_) that help elucidate the controls and formation and removal pathways of the coastal carbon cycle (Maher et al., 2015). All measurements were complemented with benthic vegetation and physicochemical data to (a) provide spatially and temporally resolved CH_4_ distribution and emission data from major northern temperate nearshore benthic environments; (b) identify potential biotic and abiotic drivers in shaping the temporal variability of CH_4_; and (c) test whether current methods are sufficient in resolving the CH_4_ distribution within highly heterogeneous and dynamic coastal settings both spatially and temporally.

## 2 MATERIALS AND METHODS

### 2.1 Study area

This study compares three distinct nearshore shallow (<4 m water depth) coastal habitats located on the island of Askö in the Baltic Sea (58°49'15.4"N 17°38'08.8"E). The habitats are representative for globally pervasive coastal ecosystems and were identified according to their dominant type of substrate and vegetation: (1) Mixed‐vegetated communities of vascular plants and algae on sediments (hereafter “mixed‐vegetated” habitat); (2) mixed turf‐ and macroalgae on rocks with pockets of sediments (hereafter “algae‐dominated” habitat), and (3) surrounding soft sediments without major macrovegetation cover (hereafter “bare sediments”). Each habitat was assessed visually, and the percent cover of the underlying substrate and macrovegetation was recorded within a 5‐m radius. Taxa that could not be identified underwater were sampled and confirmed in the laboratory. Benthic surveys were repeated in April and September 2020. Overall, the mixed‐vegetated habitat was characterized by coarse sediments with 60–90% total vegetation cover. The vegetation was dominated in equal parts by vascular plants (e.g., *Phragmites australis*, *Stuckenia pectinata*, and *Ruppia spiralis*) and benthic algae (e.g., *Chara aspera* and heterogenous assemblages of filamentous algae). The “algae‐dominated” habitat was situated on rocks and boulders with pockets of permeable sediments with 80–95% total vegetation cover comprised of the macroalgae *Fucus vesiculosus*, and *Ulva* spp., the encrusting *Hildenbrandia rubra*, and various filamentous algae. No vascular plants were identified in this habitat. The surrounding bare sediment habitat with fine soft sediments had 7–10% total vegetation, of which were mainly dislodged *F*. *vesiculosus* and filamentous algae. The study was conducted at the SW facing side of the island, which is dominated by rocky cliffs and shallow embayments and is relatively open to the Baltic Sea. The habitats were fully submerged at all times due to the absence of tides in this region of the Baltic Sea (Medvedev et al., 2016). The average of measured salinities (i.e., per sampling period and habitat) in the studied area ranged from 6.2 to 7.0 over the course of the year, and, thus, reflected brackish water conditions typical for the central Baltic Sea. While the Baltic Sea receives freshwater inflows from land and has limited saltwater inflows from the Danish straits, locally at the study site on the island in the outer Stockholm archipelago, there were no major freshwater inputs from rivers or streams, which is reflected by relatively constant salinity throughout the measurement period.

### 2.2 Experimental design

We quantified the partial pressures of surface water and atmospheric CH_4_ and CO_2_ along with the related C‐isotopes (i.e., δ^13^C‐CH_4_ and δ^13^C‐CO_2_, respectively) in the three habitats during five measurement periods in 2020 and 2021 (i.e., May 18–29; July 6–17; August 17–29; November 30 to December 8, 2020; March 1–6, 2021). For the measurements, we used an adapted version of the Water Equilibration Gas Analyzer System (WEGAS) (details in Humborg et al., 2019) coupled to a CRDS. The system consists of four major components: (i) a submersible seawater intake pump at around 0.3 m water depth mounted to a movable raft that can be deployed noninvasively over the various habitats; (ii) a water handling system comprised of a showerhead equilibrator (1 L headspace volume) and a thermosalinograph (Seabird TSG 45) fed via a hose by the seawater intake pump; (iii) a gas handling system with circulation pumps for the showerhead and ambient air; and (iv) the CRDS gas analyzer for CH_4_ and CO_2_ concentration and related C‐isotope measurements (model G2201‐i, Picarro Inc.). The use of a large seawater intake pump results in the combined measurement of CH_4_ from ebullition (bubbles) and the dissolved form in the water. The individual contribution of the two forms can, however, not be resolved using the current system. For CH_4_ and CO_2_ analyses, gas in the showerhead of the equilibrator was measured for 35 min, followed by gas measurements of ambient air for 10 min (i.e., one complete cycle was 45 min). These measurement cycles (i.e., 35 min, water; and 10 min, air measurements) ran continuously during the five measurement periods mentioned above. The raft with the water intake pump was moved between the defined habitats every 24 h from the shore with ropes. Measurements in March were distinct from the other sampling periods due to the ice cover that had been present for 4–6 weeks prior to the time of sampling. Here, holes were drilled into the ice and the pump lowered to measure “under‐ice” concentrations. We validated the CRDS analyzer's performance according to the manufacturer's instructions with “ALPHAGAZ^TM^ Stable Isotope Ratio Gases” for Picarro instruments. Specifically, before each deployment period, we injected three standards with varying CO_2_ and CH_4_ bulk concentrations, and varying δ^13^C‐CO_2_ and δ^13^C‐CH_4_ isotope values (i.e., low = 1.00 ppm CH_4_, −24.20‰ δ^13^C‐CH_4_, 250.00 ppm CO_2_, −5.00‰ δ^13^C‐CO_2_; natural = 1.77 ppm CH_4_, −48.30‰ δ^13^C‐CH_4_, 399.00 ppm CO_2_, −8.50‰ δ^13^C‐CO_2_; and high = 10.00 ppm CH_4_, −68.60‰ δ^13^C‐CH_4_, 1000.00 ppm CO_2_, −20.10‰ δ^13^C‐CO_2_). Measurements with each standard ran for 10 min, and three‐point calibration lines were constructed whose regression coefficients were used to scale the unknown sample data if needed.

Concentration and isotope measurement at 1 Hz frequency were averaged and logged every 10 s. The recorded data were filtered by removing data from the transition period between ambient air and water measurements due to the response time of CRDS to sharp changes in concentrations of CH_4_ and CO_2_. Data were also removed during improper functioning (e.g., low water flow). For this study, we used 210,059 (averaged from 2,100,590 measurements at 1 Hz) data points each for CH_4_, CO_2_, δ^13^C‐CH_4_, and δ^13^C‐CO_2_ for statistical purposes. CH_4_ concentrations in water (in ppm obtained by the CRDS) were converted to molar concentrations (i.e., CH_4_ in nM) and CO_2_ was converted to pressure units (i.e., pCO_2_ in μatm) (Humborg et al., 2019). Alongside CRDS measurements, several other environmental and meteorological variables were recorded. Surface water temperature, pH, and dissolved oxygen concentrations at the point of water intake were logged every 15 min using a multiparameter sonde (model EXO2, YSI) that was calibrated prior to each deployment. Water temperature and salinity were also recorded with every CRDS measurement with a thermosalinograph (Seabird TSG 45) that was positioned before the showerhead equilibrator. Wind data observations (wind speed and direction) and air temperature were obtained from a Metek uSonic‐3 heated 3D sonic anemometer, and a Vaisala HMP155 shielded temperature probe mounted on a 1.5‐m high meteorological mast. The mast was located at the waterline in a coastal bay, approximately 400 m to the northwest of the sampled habitats. Mean winds were adjusted to a 10‐m reference height assuming a logarithmic profile with neutral stability (Haugen, 1973):

$$U10=U+\left(\frac{u^{*}}{\kappa }\right)\times \text{log}\left(\frac{10}{z_{u}}\right)$$

where *U* is the measured wind speed at height *z_u_*, u* is the measured friction velocity by the 3D sonic anemometer, and κ is the von Karman constant (0.4).

### 2.3 Exploration of the CH_4_ distribution variability

We used a generalized linear model (GLM) to examine differences across habitats within each month. Due to positive‐skewed data and overdispersion, a quasi‐Poisson model was constructed using the glm() function in r (R Core Team, 2021) with “Month” (i.e., March, May, July, August, December) and “Habitat” (i.e., Mixed‐vegetated, Algae‐dominated, and Bare sediments) as factors. We used the R package “emmeans” (Lenth et al., 2019) for pairwise post hoc multiple comparisons with Bonferroni‐adjusted p‐values. Results and model details are presented in Table S1. The relationships among CRDS and environmental data were initially assessed using principal component analysis (PCA) using the R packages “factominer” (Husson et al., 2016) and “factoextra” (Kassambara & Mundt, 2017). PCA is a multivariate technique used to emphasize variation and to visualize patterns in a dataset, particularly when there are many variables. Upon the visual inspection of the PCA, we calculated Spearman coefficients for correlations between surface water CH_4_ concentration and potential environmental drivers (i.e., water temperature, salinity, dissolved oxygen and CO_2_ concentrations, and pH).

The thermal sensitivity of the CH_4_ distribution was further explored by applying principles of the metabolic theory of ecology (MTE) (Sibly et al., 2012), calculating the activation energy (*Ea*) based on Arrhenius equations in the seasonal thermal regime. The activation energies (*Ea* in eV) were estimated by fitting a linear regression equation between the natural logarithm of CH_4_ concentrations and the reciprocal of temperature (1/*kT*), where *k* is the Boltzmann's constant (8.62 × 10^−5^ eV K^−1^) and *T* is the measured water temperature in Kelvin. *EA*s allow for a comparison of temperature dependencies across systems and metabolic processes (Sibly et al., 2012).

We applied the Rayleigh model to estimate the fraction of CH_4_ that was oxidized in surface water in each habitat and sampling month, as:

$$\delta^{13}C_{\text{CH}4}\left(\text{CRDS}\right)=\delta^{13}C_{\text{CH}4}\left(S\right)+\varepsilon \left(\text{ln}f\right)$$

where δ^13^C_CH4_(CRDS) is the isotopic composition of surface water CH_4_ measured with the CRDS system, δ^13^C_CH4_(S) is the isotopic value of the CH_4_ source in sediments, −67‰ that has been measured in local sediments, ε is the isotope fractionation factor for CH_4_ oxidation of −20‰ (Bastviken et al., 2002), and ƒ represents the fraction of remaining CH_4_ in surface water, that is, 1−f is the fraction of oxidized CH_4_. The Rayleigh model assumes a closed system when CH_4_ oxidation occurs, which means CH_4_ oxidation is the only sink of CH_4_ in water column and is faster than the refreshment of CH_4_ supplied to the surface water. This is an oversimplification given the high variability of coastal systems. The true fraction of CH_4_ oxidized in surface water could, thus, be underestimated due to the contribution of ^13^C‐depleted CH_4_ source mixing with surface water CH_4_ with higher δ^13^C_CH4_ values in a partially open system.

### 2.4 Sampling effort evaluation of dissolved CH_4_ concentrations

We used a bootstrapping exercise to determine the minimum number of concentration measurements per day required to obtain a high‐accuracy, representative daily mean dissolved CH_4_ concentration across the various coastal habitats and sampling months. The assumption of these simulations is that our high‐resolution sampling effort (i.e., one sample per second) can sufficiently capture the temporal variations of surface water CH_4_ concentrations for each habitat. We randomly sampled the population of measured CH_4_ concentrations assuming a variable sample size, from 1 to 600 samples a day (with sample replacement). This sampling was repeated 200 times for each sample size, and for each simulation, we calculated the resulting mean CH_4_ concentration.

### 2.5 Sea–air flux computation

The sea–air flux (F) of CH_4_ was calculated as:

$$F=k\times K_{0}\times \left(pCH4_{\mathrm{sea}}‐pCH4_{\mathrm{air}}\right)$$

where *k* (m s^−1^) is the gas transfer velocity, *K*_0_ (mol m^−3^ atm^−1^) is the aqueous‐phase solubility of CH_4_, and *p*CH4_sea_ and *p*CH4_air_ are the measured partial pressures (atm) of CH_4_ in the near‐surface water and in the air, respectively. We compared our site‐specific atmospheric CH_4_ concentration measurements to data of the closest ICOS atmospheric monitoring station (i.e., Utö—Baltic Sea station; station ID: UTO). Locally measured *p*CH_4_ ranged from 1.884 to 2.124 (mean ± SE = 1.969 ± 0.063) over the study period, which compares to 1.921–2.112 (mean ± SE = 1.978 ± 0.001) from the ICOS Utö—Baltic Sea station (Laurila, 2021). Despite similar mean values over the study period, locally measured *p*CH_4_ reflects better the site‐specific variability and is, thus, more suitable for the computation of air–sea fluxes, especially if concentration gradients (i.e., water to atmosphere) are small. The solubility is determined from Wiesenburg and Guinasso (1979) as:

$$\begin{array}{c} ln\;\beta =A1+A2\left(100/T\right)+A3ln\left(T/100\right)+S\left[B1+B2\left(T/100\right)+B3{\left(T/100\right)}^{2}\right] \end{array}$$

where β is the dimensionless (mL of gas dissolved per mL of H_2_O) Βunsen solubility coefficient, A1, A2, A3, and B1, B2, B3 are constants from Table 1 in Wiesenburg and Guinasso (1979), *T* is the measured water temperature (K), and *S* the measured salinity (‰). Assuming CH_4_ behaves as an ideal gas, *K_0_* is related to β by *K_0_* = β (*R* × *T*_STD_)^−1^, where *R* (m^3^ atm K^−1^ mol^−1^) is the ideal gas constant and *T*_STD_ (K) is the standard temperature in Kelvin.

**TABLE 1:** Methane (CH_4_) concentrations and saturations in the three studied nearshore coastal habitats

Month	Habitat	CH_4_ (nM)				CH_4_ saturation (%)	*N*
		Mean (±SD)	CV (%)	Median (IQR)	Range	Median (IQR)	
March*	Mixed‐vegetated	90 (±17)	19	84 (78–96)	68–152	1659 (1530–1892)	6904
	Algae‐dominated	68 (±4)	6	67 (66–70)	57–82	1320 (1289–1371)	4495
	Bare sediments	74 (±5)	7	74 (71–76)	60–102	1438 (1389–1493)	6083
May	Mixed‐vegetated	56 (±17)	30	56 (42–69)	17–103	1369 (1034–1672)	19,573
	Algae‐dominated	41 (±15)	37	40 (27–49)	17–101	980 (731–1159)	17,894
	Bare sediments	40 (±8)	20	41 (35–45)	20–75	980 (908–1071)	18,056
July	Mixed‐vegetated	119 (±33)	28	112 (99–144)	58–204	3056 (2720–4059)	7182
	Algae‐dominated	80 (±24)	30	71 (66–85)	45–169	1949 (1800–2335)	10,885
	Bare sediments	69 (±17)	25	70 (54–82)	34–115	1977 (1517–2249)	11,961
August	Mixed‐vegetated	190 (±74)	39	174 (150–211)	53–460	5275 (4624–6563)	21,801
	Algae‐dominated	144 (±54)	38	153 (97–189)	41–274	4755 (2847–5850)	23,597
	Bare sediments	161 (±53)	33	151 (133–185)	41–324	4570 (3991–5835)	19,210
December	Mixed‐vegetated	18 (±7)	39	19 (12–24)	6–37	426 (258–526)	17,253
	Algae‐dominated	13 (±3)	23	12 (11–15)	9–23	252 (230–332)	11,878
	Bare sediments	9 (±2)	22	9 (8–10)	6–17	191 (163–214)	10,587
Annual	Mixed‐vegetated	97 (±79)	81	77 (34–143)	6–460	1707 (849–4287)	75,413
	Algae‐dominated	79 (±61)	77	65 (32–116)	9–274	1508 (803–3250)	68,749
	Bare sediments	79 (±64)	81	57 (38–122)	6–324	1424 (937–3690)	65,897

The saturation of CH_4_ is relative to the dissolved equilibrium with ambient air.

Abbreviations: CV, coefficient of variation; IQR, interquartile range; *N*, number of individual observations (10 s average of 1 Hz measurements); SD, standard deviation. The asterisk denotes under‐ice sampling in March.

The gas transfer velocity (*k*) used is that determined by Wanninkhof (2014) as:

$$k=0.251\times U^{2}\times {\left(\frac{Sc_{\mathrm{balticsea}}}{660}\right)}^{‐0.5}$$

where *U* is the wind speed (m s^−1^) at 10 m height and Sc_balticsea_ is the Schmidt number at the measurement site, which is dependent on temperature, salinity, and gas molecule. Sc was corrected for the corresponding temperature that was measured simultaneously with partial pressures of CH_4_ (*p*CH_4_) according to coefficients taken from Table 1 in Wanninkhof (2014). Furthermore, the Schmidt number for Baltic Sea brackish water (i.e., Sc_balticsea_) with measured salinity (S_balticsea_) was calculated by interpolation of Sc for fresh water (salinity 0‰) and seawater (salinity 35‰) following (Gülzow et al. 2013) and (Jähne et al. 1987):

$${\text{Sc}}_{\text{balticsea}}=\frac{\left({\text{Sc}}_{\text{seawater}}‐{\text{Sc}}_{\text{freshwater}}\right)\times S_{\text{balticsea}}}{35}+{\text{Sc}}_{\text{freshwater}}$$

All fluxes are expressed in μmol CH_4_ m^−2^ day^−1^. Other variables (e.g., currents, waves, water depth) can also be used to predict *k* in coastal environments, but the studied location does not have any significant permanent or tidal currents, and estuarine models may not provide better results for our setting. Furthermore, Lundevall‐Zara et al. (2021) tested other wind‐based k models in similar habitats of the same location and concluded that calculated average k‐values from different models were close to those of the Wanninkhof (2014) relationship for the range of wind velocities encountered on the island of Askö. Thus, for a better comparability across studies, we have therefore decided to use this relationship.

### 2.6 Estimating annual sea–air fluxes of CH₄

We estimated sea–air fluxes of CH_4_ across all habitats over the entire annual cycle. Based on the strong temperature dependencies of CH_4_ concentrations, we calculated CH_4_ concentrations outside of the measurement periods using the Arrhenius equations from Figure 3b and the 15‐min interval surface water temperature measurements from March 3, 2020 to March 3, 2021 (Figure 1b), as:

$$\ln\;{\text{CH}}_{4}\left(\text{nM}\right)=a+b*\left(\frac{1}{kT}\right)$$

with ln CH_4_ as the natural logarithm of the CH_4_ concentration in nM, a and b as intercept and slope, respectively, and the reciprocal of temperature (1/*kT*), where *k* is the Boltzmann's constant (8.62 × 10^−5^ eV K^−1^) and *T* is the measured water temperature in Kelvin. For the mixed‐vegetated, algae‐dominated, and the bare sediment habitat, the intercepts were 47.14, 47.36, and 51.66, respectively, and the slopes were −1.02, −1.03, and −1.13, respectively.

We determined the difference between the measured and the estimated CH_4_ concentrations per habitat and month (where measured data were available) as the percentage of the calculated value (i.e., the percent error). Overall, there was a good agreement of the temperature‐based calculated CH_4_ concentrations with the actual measured concentrations across all habitats in May, July, and August (i.e.; mostly <10% deviation of the means; Table S2). Calculated CH_4_ concentrations in March and December tended to be underestimated by 20–50% relative to the measured concentrations. The data show that temperature can be a good proxy to estimated CH_4_ concentrations if enough in situ data are available. However, it also becomes apparent that, when absolute concentrations are low, disparities of few nanomole in the CH_4_ concentration likely contributed to large differences (Table S2).

Sea–air fluxes of CH_4_ were then calculated based on equations provided above, assuming an average salinity of 6.6 (i.e., the average of measured salinities ranging from 6.2 to 7.0 over the course of the year). Wind speed data from the study location matching the CH_4_ concentrations (measured and calculated) was available for 21,445 out of 34,932 data points (61%). The remaining wind speed data were estimated using a Monte‐Carlo simulation on the distribution (mean ± SD, 2.25 ± 2.01 m/s) of available wind speed data from that year.

## 3 RESULTS

### 3.1 CH_4_ concentrations and δ^13^C‐CH_4_ values across coastal habitats

We report a high spatial and temporal variability of surface water CH_4_ concentrations across the mixed‐vegetated, algae‐dominated, and bare sediment habitats that span two orders of magnitude, ranging from 6 to 460 nM (Figure 1c; Table 1). During all sampling periods, the highest concentrations were always observed in the mixed‐vegetated habitat, followed by algae‐dominated, and surrounding bare sediment habitats (Table 1). A generalized linear model (GLM) with pairwise post hoc multiple comparisons confirmed that CH_4_ concentrations differed significantly across habitats within each sampling month (Table S1), with an exception of the algae‐dominated and bare sediment habitats in May. In addition, differences between the algae‐dominated and bare sediment habitats were minor (expressed by odds ratios close to 1 as effect size statistics) in May, July, and August (Table S1). There were strong seasonal variations of CH_4_ concentrations with similar patterns across all habitat types. In general, the highest CH_4_ concentrations were observed in August, followed by July, March, May, and December (Figure 1c; Table 1). The δ^13^C‐CH_4_ values of surface water varied by >7‰ over the sampling months in all habitat types. Across all habitats, CH_4_ was most enriched in ^13^C in December (average of −55‰) and became most depleted in March, approaching −63‰ (Figure 1d). Differences in δ^13^C‐CH_4_ values across habitats in the same month occurred only in some cases and were smaller than the annual temporal variation (Table S3).

**FIGURE 2:**
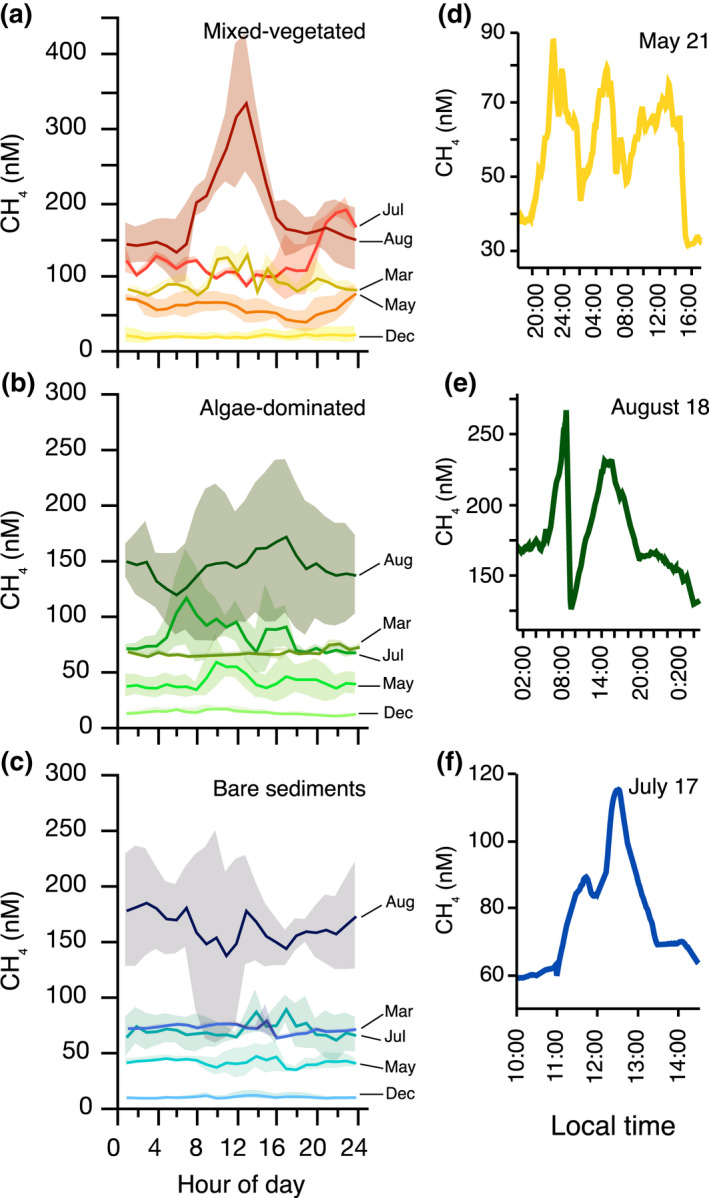
Mean of hourly CH_4_ concentrations over full diel cycles in mixed‐vegetated (a), algae‐dominated (b), and bare sediment (c) habitats during various sampling months, and exemplary CH_4_ concentration peaks (‘peak events’) of continuous (1 Hz) data in the respective habitats (d–f). Shaded areas in (a–c) depict the standard deviation around the mean derived from multiple days of measurements within the same month. Note the different scales on the y‐axes

CH_4_ concentrations also varied greatly during each sampling month and in each habitat type (Figure 1c). Most variability within months was ascribed to CH_4_ concentration changes independent of the time of the day (Figure 2a‐c). “Peak events” with >100% change of the CH_4_ concentrations occurred within hours and were observed in all habitats and during all sampling campaigns (Figure 2d‐f). We used the coefficient of variation (CV) as a standardized measure that describes the dispersion of the CH_4_ distribution around the mean to quantify and compare the within‐month variability. Overall, the CVs ranged from 5% to 39%, with the lowest variability of CH_4_ concentrations in March when the surface water was covered with ice and the highest variability generally occurring in July and August (Table 1). An exception to the seemingly random CH_4_ variability within one diel cycle was the mixed‐vegetated habitat in August, when CH_4_ consistently peaked during midday (mean ± SD, 333 ± 93 nM at 13:00 h local time), and was lowest at night (141 ± 24 nM at 02:00 h local time; Figure 2a).

### 3.2 Correlation of surface water CH_4_ with environmental variables

**FIGURE 3:**
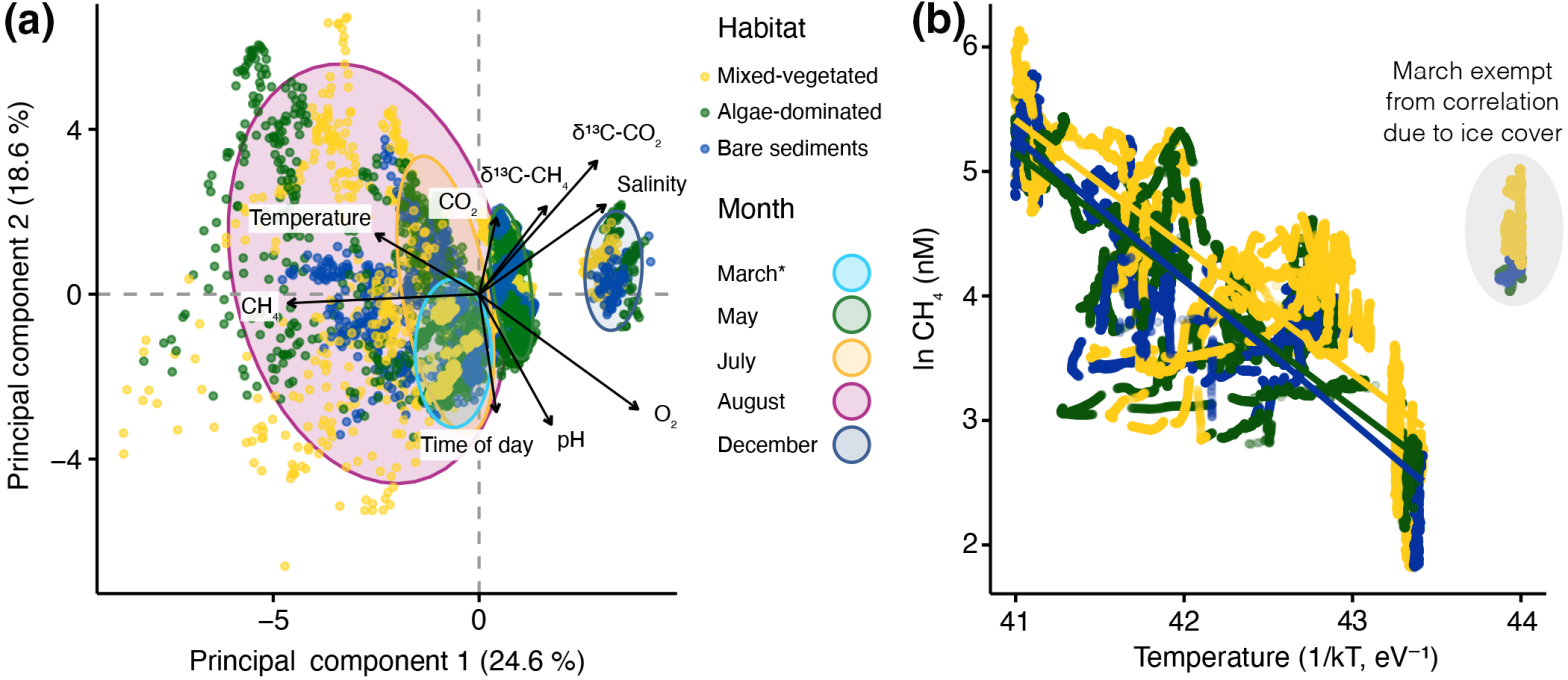
Principal component analysis (PCA) using all environmental data (a) and Arrhenius plot showing the relationship between the inverted temperature multiplied by the Boltzmann constant (1/*kT*) and the natural logarithm of the CH_4_ concentrations (b) from mixed‐vegetated, algae‐dominated, and bare sediment habitats. PCA allows the variables to be projected in multidimensional space to highlight the relationships between them. The vectors represent the individual environmental variables. When vectors are far from the center and close to each other, they are positively correlated, whereas when they are symmetrically opposed, they are negatively correlated. If the arrows are orthogonal, they are not correlated. Overall, 43.2% of the total variation is explained by the first two axes, 24.6% and 18.6%, respectively. Solid colored lines in (b) indicate the linear regression (details in the text). CH_4_ = surface water methane concentrations; CO_2_ = surface water carbon dioxide concentrations; O_2_ = surface water dissolved oxygen concentrations. The asterisk denotes under‐ice sampling in March. We excluded data points encircled in (b) from the linear regression due to ice cover in March and the resulting irregular response to temperature

Principal component analysis (PCA) revealed distinct separation of the CH_4_ and environmental data across months and to a lesser extent across habitats (Figure 3a). The first two principal components explained 43.2% of the variation in the data. Separation was greatest along principal component axis 1 (PC1 = 24.6%) that split the groups into five distinct clusters representing the sampling months March, May, July, August, and December. Variations in CH_4_ concentrations (32.1%), temperature (23.4%), salinity (14.8%), and oxygen (11.0%) contributed most to the separation of the data along principal component axis 1. Data points within each month spread predominantly along principal component axis 2 (PC2 = 18.46%), and their variation was driven by the time of the day, CO_2_ concentrations, and pH.

Upon the visual inspection of the PCA (Figure 3a), we computed correlation matrices based on Spearman's rank correlation coefficient of CH_4_ concentrations in each habitat with temperature, salinity, CO_2_, O_2_, and pH (Figure S1). Temperature showed the strongest positive association with CH_4_ concentrations in the algae‐dominated habitat (*r* ^2^(68,740) = .82, *p* < .0001), followed by bare sediments (*r* ^2^(65,812) = 0.71, *p* < .0001), and the mixed‐vegetated habitat (*r* ^2^(75,410) = 0.70, *p* < .0001). Weaker and negative associations were also apparent for O_2_, CO_2_, and salinity in all habitat types (Figure S1). pH was negatively associated with CH_4_ concentrations in the mixed‐vegetated and algae‐dominated habitat but positively in the surrounding bare sediments. Given the strong association with temperature, we further explored the thermal sensitivity of the CH_4_ distribution by calculating the apparent activation energy (*Ea* in eV) based on Arrhenius equations in each habitat within the seasonal thermal regime (Figure 1b). Estimated *EA*s were similar across all habitats with positive (i.e., higher CH_4_ concentrations at higher temperature) values of 1.13 eV (*F*(1,59,812) = 277,552.7, *p* < .0001, *r* ^2^ = .82) in bare sediments, 1.03 eV (*F*(1,64,252) = 256,516.8, *p* < .0001, *r* ^2^ = .80) in algae‐dominated, and 1.02 eV (*F*(1,65807) = 204,754.7, *p* < .0001, *r* ^2^ = .80) in the mixed‐vegetated habitat, respectively (Figure 3b).

**FIGURE 4:**
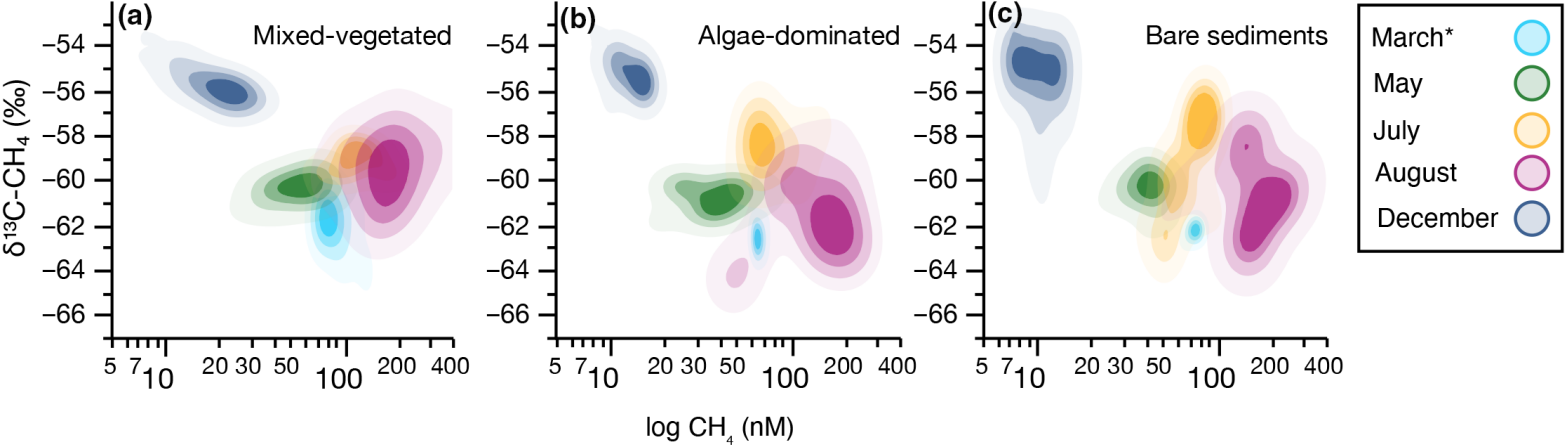
Stable carbon isotopes of methane (δ^13^C‐CH_4_) as a function of the log CH_4_ concentrations of surface water in three shallow coastal habitats (a–c) of the Baltic Sea. The data is represented as a nonparametric bivariate surface to describe the density of all data pairs (*n* = 210,059 in total). The contour lines are quantile contours in 20% intervals. The asterisk denotes under‐ice sampling in March

The δ^13^C‐CH_4_ signatures provided an additional dimension to reveal the main processes controlling CH_4_ variability given the isotope fractionation associated with CH_4_ production and consumption (i.e., oxidation) (Barker & Fritz, 1981). δ^13^C‐CH_4_ values as a function of CH_4_ concentrations reflected temporal variations across seasons (Figure 4). In all habitats, the lowest CH_4_ concentrations with the highest δ^13^C‐CH_4_ values were observed in December, while the highest CH_4_ concentrations and the lowest δ^13^C‐CH_4_ values were found in August and March. The Rayleigh model, assuming that the supply of CH_4_ is much slower than oxidation in water column, was applied to estimate the fraction of CH_4_ that was oxidized in surface water, suggesting 20% of CH_4_ loss through oxidation in August and March, and up to 50% in December in all habitats (Table S3).

### 3.3 Sea–air fluxes of CH_4_

Surface waters were supersaturated throughout all measurement periods and habitats relative to CH_4_ in ambient air. The median (IQR) CH_4_ saturation in the mixed‐vegetated habitat was 1706 (848–4286)%, 1508 (802–3250)% in algae‐dominated, and 1423 (937–3689)% in adjacent bare sediment habitats. The resulting sea–air flux rates of CH_4_ were highly variable and ranged from 0.1 to 3852 μmol CH_4_ m^−2^ day^−1^ during ice‐free periods and primarily reflected differences in water column CH_4_ concentrations between habitats and months (Table S4). The median (IQR) CH_4_ flux rates were highest during measurement periods in July, with 138 (81–245), 98 (26–172), and 77 (36–119) μmol CH_4_ m^−2^ day^−1^ in the mixed‐vegetated, algae‐dominated, and bare sediment habitat, respectively. The high CH_4_ flux rates in July coincided with high wind speeds during this month (Table S4). CH_4_ flux rates were lowest in all habitats in December, with median values of 0.7–3.5 μmol CH_4_ m^−2^ day^−1^. No fluxes were computed for the ice‐covered period. The estimated annual median (IQR) sea–air fluxes were 12 (3–43), 10 (2–32), and 7 (2–29) μmol CH_4_ m^−2^ day^−1^ in the mixed‐vegetated, algae‐dominated, and bare sediment habitat, respectively.

## 4 DISCUSSION

Our high‐resolution measurements revealed differences in the distribution of surface water CH_4_ concentrations across neighboring coastal habitats over short spatial (within meters) scales and exceptionally high temporal variability that could only be detected with continuous measurement techniques during several seasons. Acknowledging this high spatiotemporal variability is critical to confine CH_4_ emissions from coastal environments and the variability associated with their habitat heterogeneity.

### 4.1 Temperature sensitivity of coastal CH_4_ distribution

Median CH_4_ concentrations measured across the here‐studied habitats were 4–13 times higher than those observed in deeper waters of the open Baltic Sea (Schmale et al., 2010; Wilson et al., 2018), up to three times higher than previously published data for coastal Baltic areas (Humborg et al., 2019; Ma et al., 2020), and substantially higher than globally compiled nearshore CH_4_ concentrations (Weber et al., 2019) (Table S5). The magnitude highlights that vegetated coastal ecosystem are characterized by excessive organic matter loads from primary production, trapping and accumulation of allochthonous organic matter, and sedimentary conditions that can favor CH_4_ production (Dale et al., 2019; Wallenius et al., 2021). However, we also report an exceptionally high spatiotemporal variability of surface water CH_4_ concentrations.

A first major source of this variability was attributed to seasonal differences in CH_4_ concentrations. The significant correlation between CH_4_ concentrations and temperature over the sampling months suggests that temperature mainly regulates seasonal variations. Like most other forms of metabolism, methanogenesis is temperature‐dependent, and the calculated apparent activation energies (*EA* = ~1 eV, across all habitats) were in line with previous global estimates of ecosystem‐scale CH_4_ fluxes with an *EA* of 0.96 eV (Yvon‐Durocher et al., 2014). Thus, the higher CH_4_ concentrations in late summer are likely due to increased production under warmer water temperatures. Historical data from the nearby oceanographic observation station “2507 Landsort Norra” between 2010 and 2020 confirmed that the annual surface water temperature curve from our study area is representative of previous years (Sveriges meteorologiska och hydrologiska institut, 2022). We infer that the observed temperature sensitivity is primarily driven by natural temperature variations rather than a warming effect. Both aerobic CH_4_ oxidation together with anaerobic CH_4_ oxidation in sediments may also increase in summer due to temperature controlling their rates (Treude et al., 2005; Zehnder & Brock, 1980) and the increased supply of CH_4_ supply by molecular diffusion. However, in summer, the overall production of sedimentary CH_4_ likely outweighed the relative contribution CH_4_ oxidation pathways. In support of this, parallel measured δ^13^C‐CH_4_ values combined with the Rayleigh model revealed that the relative contribution of CH_4_ production versus oxidation shifted across seasons. CH_4_ oxidizing bacteria favor isotopically lighter CH_4_, leaving the residual CH_4_ with heavier isotopes. Low CH_4_ concentrations accompanied by high δ^13^C‐CH_4_ values suggest that up to 50% of CH_4_ was oxidized in winter, indicating an important role of CH_4_ oxidation in removing CH_4_ relative to CH_4_ production. This microbial oxidation efficiency decreased to 20–30% in summer due to a boosted supply of CH_4_ to the water column relative to its oxidation. High oxygen concentrations mediated by the photosynthetic activity of algae and plant communities during the day and increased light exposure in summer may have contributed to inhibiting CH_4_ oxidation (Murase & Sugimoto, 2005; Rudd et al., 1976).

### 4.2 Ice‐cover effects on CH_4_ dynamics

An exception to the overall seasonal trend was observed in March (i.e., late winter/early spring). Measurements during this month were marked by ice cover that, to this point, had been present for 4–6 weeks. Analogous to many northern lakes (Denfeld et al., 2018), we observed an accumulation of CH_4_ under the ice, with mean concentrations six times higher than in December (last month without ice cover). More negative δ^13^C‐CH_4_ values in March (−62 to −64‰) suggest CH_4_ supply with overall low oxidation. This observation corroborates studies showing suppressed methanotrophic activity at very cold temperatures (e.g., Phelps et al., 1998). Calculations of the Rayleigh model confirmed that <20% of the surface water CH_4_ was oxidized during this period. However, the CH_4_ depleted in ^13^C could also be a result of varying fractionation during methanogenesis at lower temperatures or mixed CH_4_ formation pathways. The CH_4_ accumulation under ice will likely result in enhanced outgassing events following ice break (Ducharme‐Riel et al., 2015; Karlsson et al., 2013). Whereas under‐ice CH_4_ accumulation is a well‐studied feature of northern lakes, these dynamics have not been described for northern temperate coastal regions with regular sea ice every year. Our data suggest the necessity to include the ice‐covered period and CH_4_ outgassing during ice breakup in future coastal CH_4_ sampling strategies and the annual CH_4_ budget of northern temperate and high‐latitude regions (Omstedt et al., 2004).

### 4.3 Physical forcing may drive short‐term CH_4_ variability

A second major source of variability in the CH_4_ concentrations was short‐term variations that occurred within hours (Figure 2d–f). Most of this variability was independent of the time of the day and without an apparent and reoccurring diel pattern. However, fluctuations of the CH_4_ concentrations were so strong that the minimum and maximum values within one habitat and sampling campaign (time window max. 12 days) could differ by up to one order of magnitude (Table 1). The dispersion of the CH_4_ probability distribution around the mean concentration was on average 30% during the ice‐free months and, thus, much higher than the reported global open ocean CH_4_ variability with CVs ranging between 2% and 11% (Wilson et al., 2018). While we could not find any direct correlation to the available environmental data, one possible explanation for the high variability could be the physical influence of the open coastal setting through wind and/or wave action. A wave‐induced pumping effect on the pore water pressure can transport solutes from deeper to surface layers (Precht & Huettel, 2004; Yang et al., 2019); Thus, varying CH_4_ release rates from permeable coastal sediments in very shallow waters may cause variable near‐surface CH_4_ concentrations, as has been shown relevant even for lake systems (Hofmann et al., 2010). In support of this, the CVs of the CH_4_ distribution were much lower across all habitats in March (mean CV = 10%), when, due to ice cover, the influence of waves and winds on the water column and sediments was likely minor and no CH_4_ escaped to the atmosphere.

### 4.4 Reoccurring diel CH_4_ patterns in summer

A reoccurring diel pattern in CH_4_ concentration changes was only observed in the mixed‐vegetated habitat in August, with the highest concentrations consistently toward midday and lowest at night. This marked diel variation may be attributed to plant‐mediated transport of CH_4_ by convective throughflow from rooted submerged plants, which were only present in the mixed‐vegetated habitat. The convective transport through pressure gradients can account for up to 60% of the total CH_4_ transport from sediments during daylight hours and high photosynthetic activity (Kim et al., 1998; van den Berg et al., 2020). In the early stages of plant growth, molecular diffusion through dead/live plants into the standing water column can be the primary transport mechanism (Kim et al., 2001). Most plants at the mixed‐vegetated site were fully submerged; thus, a sediment–plant–water flux is likely. However, *Phragmites* stems (comprising ~10% of the total vegetation in the mixed‐vegetated site) possibly facilitated a sediment–plant–air flux of CH_4_ (van den Berg et al., 2020), which will have remained undetected with our approach. Abiotic CH_4_ photoproduction from organic matter degradation may also play a role in shaping site‐specific CH_4_ dynamics in oxygenated surface waters (Li et al., 2020; Zhang & Xie, 2015). However, given that reoccurring and pronounced diel cycles were only visible in one of the three neighboring habitats, benthic/plant‐mediated pathways seem more likely to have caused the patterns observed. Overall, the contribution of plant‐mediated fluxes and the relation to seasonal succession patterns of submerged vegetation, along with the contribution of CH_4_ photoproduction in shallow coastal waters with high incident irradiance remain uncertain and need further investigation.

### 4.5 Spatial distribution of CH_4_ reflects coastal ecosystem mosaic

Shallow coastal habitats are heterogeneous, and the variation in spatial structure and temporal change of benthic communities defines the expression of ecosystem functions in form and magnitude (Snelgrove et al., 2014). Reflecting the coastal ecosystem mosaic (Sheaves, 2009), some of the measurements across the studied neighboring habitats were not further than 30–50 m apart. Yet, despite their proximity, we observed significant differences in the distribution of CH_4_ in the water column and the magnitude of the resulting sea–air fluxes. Surface water CH_4_ concentrations are likely related to variable CH_4_ production and oxidation rates, as indicated by varying δ^13^C‐CH_4_ values across sites during some months (Figure S3). These differences may be ascribed to different quantities and qualities of organic matter deposited within local sediments and differences of sediment properties (e.g., porosity) (reviewed in Rosentreter et al., 2021a). The presence of rooted vegetation may also play a role in the small‐scale variability, as roots provide substrate via root litter and exudates and transport oxygen into the sediments. In addition, while the employed system measures CH_4_ in the dissolved form and from ebullition (bubbles), the individual contribution of the two phases cannot be resolved but may contribute to differences between the habitat types. It becomes apparent that more research is required to determine the spatial scale of this variability and to understand better the controls on substrate availability for methanogenesis. In particular, links between biodiversity metrics (i.e., abundance and biomass) of primary and secondary producers and CH_4_ production and consumption pathways need to be better constrained as has been shown relevant for seafloor metabolism (i.e., gross primary production and community respiration) in shallow waters (Rodil et al., 2021). Likewise, integrating knowledge on the structure of sediment microbial communities associated with the different habitats is imperative to improve the prediction of CH_4_ production and oxidation pathways from different coastal habitats (Wallenius et al., 2021).

### 4.6 High sampling intensity is required to capture coastal CH_4_ variability

**FIGURE 5:**
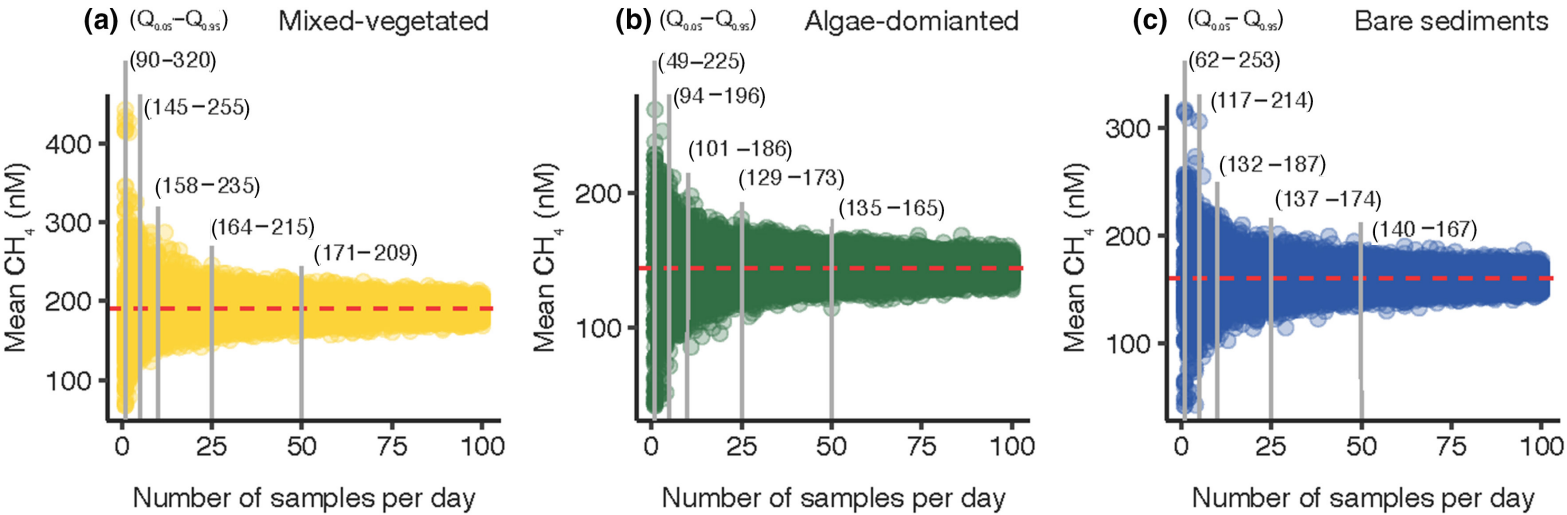
The mean surface water CH_4_ concentration obtained by bootstrapping the population of measured CH_4_ concentrations, with a sampling number ranging from 1 to 100 samples per day, and 200 replicates for each number of samples. The numbers in parentheses show the 5th–95th percentiles [Q_0.05_–Q_0.95_] for 5, 10, 25, and 50 samples. The red dashed line shows the true daily mean of the measured CH_4_ concentrations. Bootstrapping results are shown for of the mixed‐vegetated (a), algae‐dominated (b) and bare sediment (c) habitat in August. Bootstrapping results of all other months in Table S5

Particularly the high temporal variability on timescales from hours to days complicates our ability to generalize the distribution of CH_4_ in nearshore coastal environments and obstructs efforts to confine diffusive flux calculations that are based on concentration measurements. Therefore, we conducted a bootstrapping analysis on our continuous data to determine the minimum number of individual concentration samples per day required to obtain a high accuracy, representative mean dissolved CH_4_ concentrations. The data exploration shows that collecting one discrete water sample a day, a typical approach used to describe CH_4_ concentration differences across geolocations (Banerjee et al., 2018; Dutta et al., 2015; Nirmal Rajkumar et al., 2008), results in a large uncertainty, with a potential to over‐ or underestimate the mean CH_4_ concentration by almost 70%. Specifically, taking only one sample per day from the mixed‐vegetated habitat in August would result in a mean CH_4_ concentration with a 5th–95th percentile of 90–320 nM based on 200 simulations. Increasing the sampling intensity to five samples per day reduces the uncertainty to 30%. In comparison, 50 samples per day instead would narrow this uncertainty to a 5th–95th percentile of 171–209 nM (10% uncertainty), closer to the observed true mean CH_4_ concentration of 191 nM during this period (Figure 5a). A similar pattern was apparent in all other habitats and sampling periods (Table S6). Consequently, the data collection and sampling strategy are detrimental to accurately capturing the temporal variability and assure justified mean CH_4_ concentrations that are the basis for flux computations. Thus, near‐continuous measurements using CRDS (Hartmann et al., 2018; Humborg et al., 2019; Maher et al., 2013) or similar systems to determine in situ CH_4_ concentrations in surface waters are desirable when addressing complex pathways and transformations of CH_4_ in coastal ecosystems. For annual estimates, seasonal measurements that reflect local climatological patterns will be required.

### 4.7 Northern temperate coastal habitats are seasonal CH_4_ emission hotspots

The high‐resolution CH_4_ concentration measurements allowed us to establish annual CH_4_ emission estimates across all habitat types. The diffusive CH_4_ fluxes suggest that northern temperate coastal habitats with mixed vegetation, algal dominance, and their adjacent bare sediment areas are net sources of atmospheric CH_4_ throughout the year.

As a result of extended periods of low temperature and temporal ice cover, the median annual fluxes were at the lower end compared to coastal wetland and tidal flat CH_4_ emissions globally (Rosentreter et al., 2021b). However, in summer, CH_4_ emissions of ≥100 μmol CH_4_ m^−2^ day^−1^ across all habitats were comparable to, or even higher than, those reported from similar (Lundevall‐Zara et al., 2021) or other vegetated coastal ecosystems (Al‐Haj & Fulweiler, 2020; Rosentreter et al., 2021b). During these periods, large amounts of carbon are turned over in the habitats studied here (Attard et al., 2019a, 2019b), and macrophyte tissues become a direct component of local sediment organic matter pools (Marcelina et al., 2018) that favor local CH_4_ production (Dale et al., 2019; Wallenius et al., 2021). Despite these seasonally relevant CH_4_ emissions, there is still a paucity of data from northern temperate coastal habitats in general, and they are exceedingly underrepresented in current global CH_4_ budgets. Yet, just in the Baltic Sea, the potential distribution area in waters of <5 m depth is almost 30.000 km^2^ (HELCOM, 2013; Jakobsson et al., 2019), and, thus, equals 22% of the global areal extent of mangroves (Bunting et al., 2018) or 19% that of seagrass meadows (McKenzie et al., 2020). Thus, we: (a) postulate that nearshore habitats in northern temperate regions are understudied but seasonally relevant emitters of CH_4_ to the atmosphere; (b) encourage including these habitats in future coastal CH_4_ emission estimates, while also recognizing their pronounced seasonality; and (c) hypothesize that including these habitats amplifies the global ocean CH_4_ budget significantly, especially when considering that macroalgae habitats alone contribute most (>50%) to the total global extent of coastal vegetation (Duarte et al., 2013).

### 4.8 Uncertainties in coastal CH_4_ distribution and future research directions

Variations of surface water CH_4_ concentrations and resulting sea–air fluxes reflecting the heterogeneous nature of coastal environments currently complicate generalizing regional patterns and upscaling attempts globally. Given the CH_4_ distribution patterns identified in this study, we encourage several aspects to be considered to refine large‐scale coastal CH_4_ emission budgets.

First, studies currently used for global coastal CH_4_ budgets have a site‐selective bias due to their particular relevance in providing a service (e.g., they are interesting from a blue carbon perspective) and for other practical reasons like the accessibility of the study area. Here, we provided evidence that northern temperate coastal habitats, which are presently understudied for their contribution to CH_4_ fluxes (e.g., algal communities on rocky shores), can be seasonally relevant sources of atmospheric CH_4_. Similar measurements should be extended to additional coastal environments and geolocations to confirm the global relevance of their CH_4_ emissions. The spatial heterogeneity of coastal habitats provides an opportunity for measurements along environmental gradients, with great potential to increase inference across scales (Snelgrove et al., 2014).

Second, new technical approaches have to be embraced to better understand the high temporal variability of the CH_4_ distribution and the underlying processes in coastal environments. The use of continuous rather than time‐averaged measurements helps to account for short‐term temporal variations by diel cycles or peak events (Call et al., 2015; Maher et al., 2013; Rosentreter et al., 2018), and reduces uncertainties when establishing diel budgets. The high‐resolution measurements across multiple seasons and the identification of dependencies on environmental variables have also bearings for predicting future CH_4_ emissions under various changing environmental conditions.

Third, net annual CH_4_ fluxes are influenced by temporal variations throughout the year. Thus, to increase confidence when compiling data for global coastal CH_4_ budgets, better seasonal coverage of coastal CH_4_ needs to be combined with the recognition that reported mean values (both CH_4_ concentrations and emissions) might be biased toward sampling in a particular period only. As the seasonal behavior of CH_4_ is highly site‐specific, the variations need to be considered for each habitat type and geolocation.

Lastly, measurements of CH_4_ emission from northern temperate and high‐latitude coastal habitats should be acknowledged in future emission budgets. Climate change occurs particularly fast in northern hemisphere mid‐latitude (Cohen et al., 2014) and high‐latitude (Screen & Simmonds, 2010; Serreze et al., 2009) regions. Specifically, as Earth approaches an average warming of 2°C, some northern hemisphere high‐latitude regions are expected to reach 4°C annual warming, outpacing the global average (Overland et al., 2014; Post et al., 2019). Although we could show a nonlinear behavior of CH_4_ emissions with temperature, future studies aiming at resolving questions associated with climate change need to consider inter‐annual rather than seasonal variations in CH_4_ emissions and the balance of all important carbon pathways that influence CH₄ production pathways (Yvon‐Durocher et al., 2014).

## 5 CONCLUSION

We conducted seasonal sampling campaigns of dissolved CH_4_ concentrations and δ^13^C‐CH_4_ values using a fast‐response automated gas equilibrator and CRDS system across three globally pervasive vegetated and nonvegetated coastal habitats. As the first study to compare high‐resolution measurements across neighboring habitats, we highlight unprecedented spatiotemporal variability of the CH_4_ distribution driven by habitat‐specific CH_4_ production and consumption pathways, seasonal temperature dependencies, and short‐term fluctuations. A bootstrapping analysis on the continuous data revealed that scaling the CH_4_ distribution from few samples involves large errors, and at least ~50 samples per day are needed to achieve accurate emission estimates. Failing to include such high‐resolution measurements in future global CH_4_ assessments may result in a continued systematic bias of regional and global estimates due to the lack of measurements representative for the coastal ecosystem mosaic—a highly heterogenous environment in space and time. Ultimately, a better understanding of the habitat‐specific contribution to the global CH_4_ emission budget would improve efforts to address climate change, such as by revealing the net potential of coastal blue carbon habitats to sequester carbon.

## CONFLICT OF INTEREST

The authors declare that they have no competing interests.

## AUTHOR CONTRIBUTIONS

F.R., C.H., and A.N. involved in conceptualization; F.R., X.S., M.C.G., and C.H. involved in methodology; C.H., A.N., J.P., and V.B. involved in material and logistics; F.R., X.S., J.P., S.B., E.B., F.N., and C.H. investigated the study; F.R., X.S., J.P., C.H., and A.N. contributed in data interpretation and data analysis; F.R., X.S., C.H., and A.N. wrote the original draft; all authors reviewed and edited the manuscript.

## Supporting information

Supplementary Material
