# Supplementary material for: High spatiotemporal variability of methane concentrations challenges estimates of emissions across vegetated coastal ecosystems
Source: Glob Chang Biol. 2022 Apr 12;28(14):4308–22. doi: 10.1111/gcb.16177 (PMC9540812; doi:10.1111/gcb.16177)
Supplement: Supplementary file 1 — Supplementary Material [file GCB-28-4308-s001.docx]

**Supplementary Information for**

**High spatiotemporal variability of methane concentrations challenges estimates of emissions across vegetated coastal ecosystems**

Florian Roth^1,2^*, Xiaole Sun^3,1^, Marc C. Geibel^1^, John Prytherch^4^, Volker Brüchert^5,6^, Stefano Bonaglia^7^, Elias Broman^1,8^, Francisco Nascimento^1,8^, Alf Norkko^1,2^, Christoph Humborg^1,2^

^1^ Baltic Sea Centre, Stockholm University, Stockholm, Sweden.

^2^ Tvärminne Zoological Station, University of Helsinki, Hanko, Finland.

^3^ Center of Deep Sea Research, Institute of Oceanology, Chinese Academy of Sciences, Qingdao, China.

^4^ Department of Meteorology, Stockholm University, Stockholm, Sweden.

^5^ Department of Geological Sciences, Stockholm University, Stockholm, Sweden.

^6^ Bolin Centre for Climate Research, Stockholm University, Stockholm, Sweden.

^7^ Department of Marine Sciences, University of Gothenburg, Gothenburg, Sweden.

^8^ Department of Ecology, Environment and Plant Sciences, Stockholm University, Stockholm, Sweden.

*Corresponding author

**Email:** florian.roth@su.se

**This file includes:**

Figures S1 to S2

Tables S1 to S6


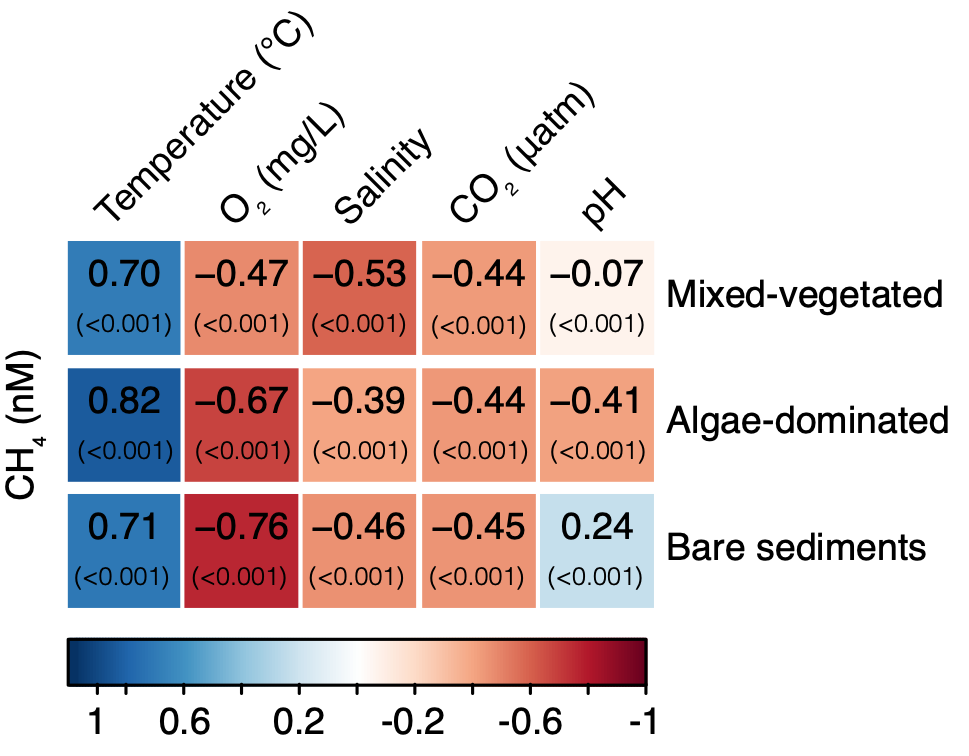


Fig. S1. Spearman's rank correlation coefficients of CH_4_ concentrations in each habitat with temperature, O_2,_ salinity, CO_2_, and pH. Significant levels are shown in brackets. Darker colors denote a stronger and lighter colors a weaker correlation. Blue are positive and red negative correlations.


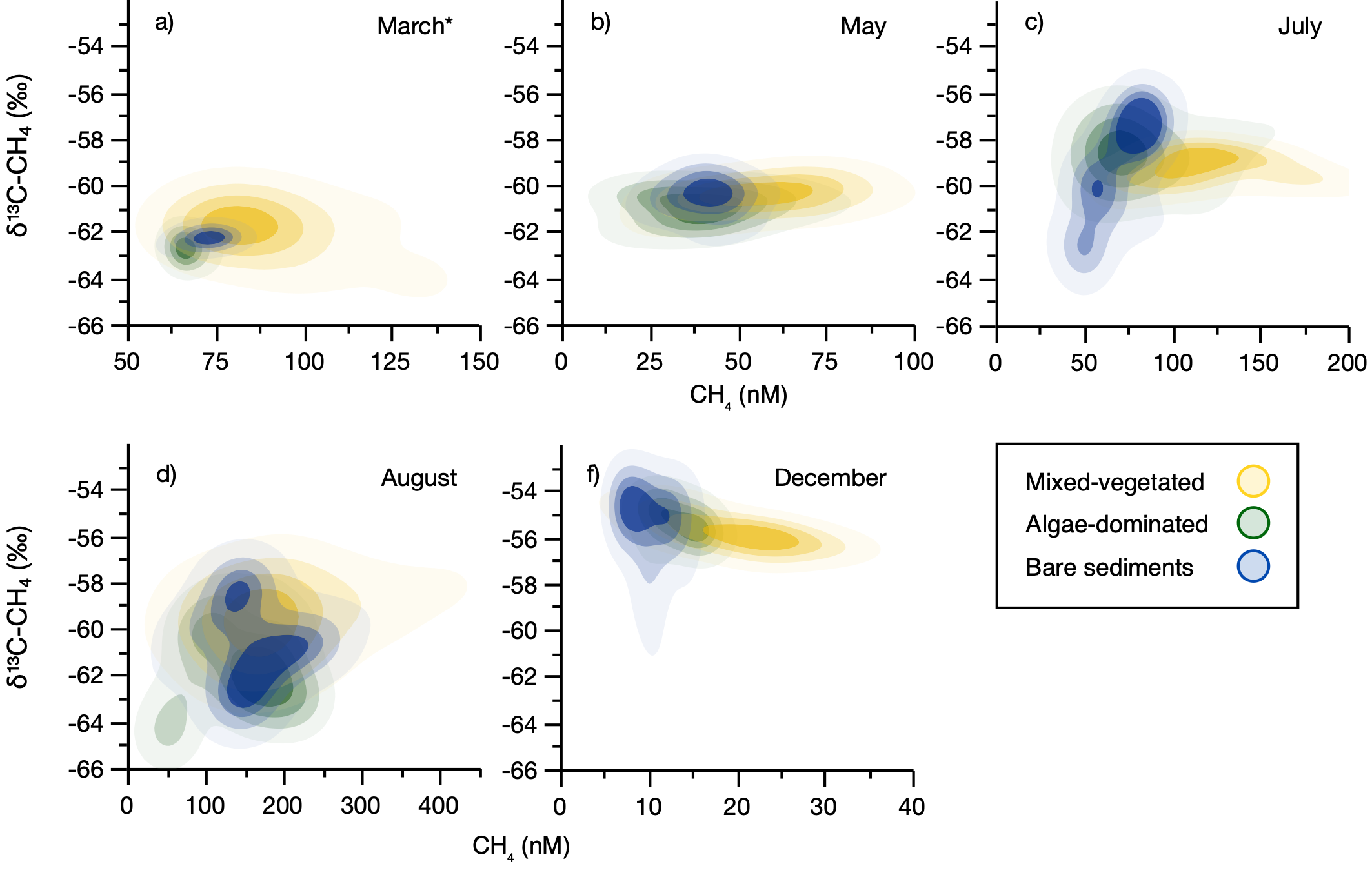


Fig. S2. Stable carbon isotopes of dissolved methane (δ^13^C-CH_4_) as a function of the CH_4_ concentrations of surface water during five sampling months (a-f) in three coastal habitats of the Baltic Sea. The data is represented as nonparametric bivariate surface to describe the density of all data pairs (n = 210,059 in total). The contour lines are quantile contours in 20% intervals. The asterisk denotes under-ice sampling in March.

| **Model info** | |
| --- | --- |
| **Info** | **Value** |
| Model Type | Quasi-Poisson |
| Call | glm(CH4 ~ Month + Habitat + Month:Habitat) |
| Link function | log |
| R-squared | 0.823 |
| AIC | NaN |
| BIC | NaN |
| Deviance | 1.93E+06 |
| Residual DF | 210044 |
| Chi-squared/DF | 9.235 |
| Converged | yes |

| **Loglikelihood ratio tests** | |  |  |
| --- | --- | --- | --- |
|  | **X²** | **df** | **p** |
| Month | 921156 | 4 | < .001 |
| Habitat | 25466 | 2 | < .001 |
| Month*Habitat | 7410 | 8 | < .001 |

| **Post Hoc Comparisons - Month ✻ Habitat** | | | | | | | | | |
| --- | --- | --- | --- | --- | --- | --- | --- | --- | --- |
| **Comparison** | | | | | |  | | | |
| **Month** | **Habitat** |  | **Month** | **Habitat** | **exp(B)** | | **SE** | **z** | **p_bonferroni_** |
| March | Algae-dominated | – | March | Mixed-vegetated | 0.7564 | | 0.00484 | -43.59 | < .001 |
| March | Algae-dominated | – | March | Bare sediments | 0.922 | | 0.00658 | -11.38 | < .001 |
| March | Mixed-vegetated | – | March | Bare sediments | 1.2189 | | 0.00683 | 35.35 | < .001 |
| May | Algae-dominated | – | May | Mixed-vegetated | 0.7267 | | 0.00333 | -69.6 | < .001 |
| May | Algae-dominated | – | May | Bare sediments | 1.0082 | | 0.00507 | 1.63 | 1 |
| May | Mixed-vegetated | – | May | Bare sediments | 1.3875 | | 0.00636 | 71.4 | < .001 |
| July | Algae-dominated | – | July | Mixed-vegetated | 0.6705 | | 0.0031 | -86.46 | < .001 |
| July | Algae-dominated | – | July | Bare sediments | 1.1524 | | 0.00537 | 30.44 | < .001 |
| July | Mixed-vegetated | – | July | Bare sediments | 1.7186 | | 0.00804 | 115.75 | < .001 |
| August | Algae-dominated | – | August | Mixed-vegetated | 0.7588 | | 0.00169 | -124.19 | < .001 |
| August | Algae-dominated | – | August | Bare sediments | 0.8965 | | 0.00214 | -45.77 | < .001 |
| August | Mixed-vegetated | – | August | Bare sediments | 1.1815 | | 0.0027 | 73.03 | < .001 |
| December | Algae-dominated | – | December | Mixed-vegetated | 0.6847 | | 0.00653 | -39.69 | < .001 |
| December | Algae-dominated | – | December | Bare sediments | 1.3596 | | 0.017 | 24.57 | < .001 |
| December | Mixed-vegetated | – | December | Bare sediments | 1.9856 | | 0.02207 | 61.7 | < .001 |

Table S1. Results of generalized linear model (GLM) comparing methane (CH_4_) concentrations across habitats in each sampling months. exp(B), or the odds ratio, is the predicted change in odds for a unit increase in the predictor. z value is the Wald statistic. p_bonferroni_ is the p-value with Bonferroni correction. The Post Hoc results only show the pairwise comparison of habitats within each month.

| **Habitat** | **March** | **May** | **July** | **August** | **December** |
| --- | --- | --- | --- | --- | --- |
| Mixed-vegetated | -53.2 ± 4.3% | -5.5 ± 6.5% | -9.2 ± 3.0% | -11.0 ± 2.3% | -24.1 ±1.0% |
| Algae-dominated | -41 ± 3.8% | -3.6 ± 3.5% | -4.6 ± 3.8% | -10.9 ± 2.1% | -44.8 ±1.0% |
| Bare sediments | -32.6 ± 3.2% | -11.1 ± 2.3% | 11.7 ± 2.2% | -6.7 ± 2.9% | -58.9 ±0.9% |

**Table S2.** Differences (%) of the measured and calculated surface water CH_4_ concentrations for estimating annual sea-air fluxes of CH_4_ based on seasonal in situ measurements. Presented is the percent error as the difference between the measured and the estimated CH_4_ concentrations per habitat and month (where measured data was available) as mean ± SE. Negative values correspond to an underestimation and positive values correspond to an overestimation of the calculated value relative to the measured concentrations.

|  |  | **δ^13^C-CH₄**  **(in ‰)** |  | **Fraction of oxidized surface water CH₄ (in %)** |  |
| --- | --- | --- | --- | --- | --- |
| **Month** | **Habitat** | Mean (±SD) |  | Mean (±SD) | N |
| March* | Mixed-vegetated | -62.6 (±0.5) |  | 22 (±0.04) | 4495 |
|  | Algae-dominated | -61.9 (±1.5) |  | 20 (±0.02) | 9604 |
|  | Bare sediments | -62.3 (±0.3) |  | 21 (±0.01) | 6083 |
| May | Mixed-vegetated | -60.9 (±0.7) |  | 28 (±0.02) | 17894 |
|  | Algae-dominated | -60.4 (±0.7) |  | 27 (±0.02) | 19573 |
|  | Bare sediments | -60.4 (±1.1) |  | 28 (±0.03) | 18056 |
| July | Mixed-vegetated | -58.7 (±1.7) |  | 32 (±0.02) | 10885 |
|  | Algae-dominated | -59.2 (±0.7) |  | 24 (±0.03) | 7182 |
|  | Bare sediments | -59.3 (±2.2) |  | 31 (±0.08) | 11961 |
| August | Mixed-vegetated | -61.7 (±1.6) |  | 31 (±0.05) | 23597 |
|  | Algae-dominated | -59.5 (±1.4) |  | 23 (±0.06) | 21801 |
|  | Bare sediments | -60.6 (±1.8) |  | 22 (±0.06) | 19210 |
| December | Mixed-vegetated | -54.6 (±1.3) |  | 44 (±0.03) | 11878 |
|  | Algae-dominated | -55.4 (±1.2) |  | 46 (±0.04) | 17253 |
|  | Bare sediments | -53.9 (±1.9) |  | 48 (±0.05) | 10587 |
| Annual | Mixed-vegetated | -59.9 (±2.9) |  | 31 (±0.04) | 68749 |
|  | Algae-dominated | -59.1 (±2.4) |  | 30 (±0.06) | 75413 |
|  | Bare sediments | -59.4 (±3.0) |  | 30 (±0.04) | 65897 |

Table S3. Mean isotopic composition of surface water CH_4_ (δ^13^C-CH₄) and calculated fraction (expressed in %) of CH_4_ oxidized based on a Rayleigh fractionation model. We assumed an isotope fractionation factor (ε) of 20‰ and a source δ^13^C-CH_4_ value of -67‰. Abbreviations: SD = standard deviation; N = number of individual observations (2 min running average of 1Hz measurements). The asterisk denotes under-ice sampling in March.

|  |  | **CH_4_ flux (μmol m^-2^ d^-1^)** | |  | **Wind speed (m/s)** | |  |
| --- | --- | --- | --- | --- | --- | --- | --- |
| **Month** | **Habitat** | **Median** | **IQR** |  | **Median** | **IQR** | **N** |
| March | Mixed-vegetated | - | - |  | - | - | - |
|  | Algae-dominated | - | - |  | - | - | - |
|  | Bare sediments | - | - |  | - | - | - |
| May | Mixed-vegetated | 9.36 | 2.09 - 24.63 |  | 2.02 | 1.02 – 3.27 | 19573 |
|  | Algae-dominated | 3.77 | 0.93 – 13.37 |  | 1.62 | 0.78 – 2.85 | 17894 |
|  | Bare sediments | 2.05 | 0.73 – 8.19 |  | 1.14 | 0.68 – 2.34 | 18056 |
| July | Mixed-vegetated | 137.92 | 81.15 – 244.82 |  | 4.87 | 3.95 – 6.16 | 7182 |
|  | Algae-dominated | 97.80 | 25.63 – 171.97 |  | 5.30 | 2.26 – 6.85 | 10885 |
|  | Bare sediments | 76.67 | 36.24 – 118.84 |  | 4.66 | 3.67 – 5.74 | 11961 |
| August | Mixed-vegetated | 85.28 | 18.38 – 132.71 |  | 3.00 | 1.84 – 3.00 | 21801 |
|  | Algae-dominated | 68.93 | 7.31 – 106.64 |  | 3.00 | 1.31 – 3.00 | 23597 |
|  | Bare sediments | 81.00 | 30.21 – 197.05 |  | 3.00 | 2.07 – 4.21 | 19210 |
| December | Mixed-vegetated | 3.49 | 1.19 – 6.77 |  | 2.40 | 1.47 – 4.19 | 17253 |
|  | Algae-dominated | 0.67 | 0.11 – 2.01 |  | 1.43 | 0.64 – 2.39 | 11878 |
|  | Bare sediments | 1.39 | 0.78 – 2.92 |  | 2.80 | 2.18 – 3.92 | 10587 |
| Annual* | Mixed-vegetated | 11.75 | 2.83 – 42.99 |  | 2.87 | 1.47 – 4.66 | 34932 |
|  | Algae-dominated | 9.51 | 2-31 – 32.29 |  | 2.87 | 1.47 – 4.66 | 34932 |
|  | Bare sediments | 7.00 | 1.60 – 29.03 |  | 2.87 | 1.47 – 4.66 | 34932 |

Table S4. Water-atmosphere CH_4_ flux rates and wind speeds in three northern temperate nearshore coastal habitats. Due to ice cover, no fluxes were computed for March. *The annual CH_4_ flux rates were estimated based on the available data from the five sampling periods and interpolated CH_4_ concentrations based on the strong temperature dependences of the CH_4_ concentrations (see methods for details). IQR = Interquartile range.

|  | **Median CH_4_ concentrations** |
| --- | --- |
|  | (nM) |
| **This study** |  |
| Mixed-vegetated | 19 - 174 |
| Algae-dominated | 12 - 153 |
| Bare sediments | 9 - 151 |
| **Open Baltic Sea** |  |
| Schamel et al. 2010 (1) | 3 - 12 |
| Wilson et al. 2018 (2) | 4 - 6 |
| Gülzow et al. 2013 (3), Arkona Basin | 3.2 – 8** |
| Gülzow et al. 2013 (3), Gulf of Finland | 3.4 – 22** |
| Gülzow et al. 2013 (3), Gotland Basin | 3.3 – 4.6** |
| Dzyuban et al. 1999 (4), Gotland Basin | 4.5 – 120** |
| **Coastal Baltic Sea** |  |
| Humborg et al. 2019 (5) | 20 - 47 |
| Ma et al. 2020 (6) | 51* |
| Geilfus et al 2021 (7), Bothnian Bay, winter measurements | 6.8* |
| Lundevall-Zara et al. 2020 (8), spring to fall measurements | 59 – 712* |
| **Coastal North Sea** |  |
| Borges et al. 2016 (9) | 139* |
| **Global coastal ocean** |  |
| Weber et al. 2019 (10) | 0.7 - 20 |

Table S5. Surface water CH_4_ concentrations in coastal and offshore marine environments. Ranges represent different sampling periods. Values with one asterisk (i.e., *) are mean concentrations and may, thus, be higher than the median values. Values with two asterisks (i.e., **) depict the full range of measured CH_4_ concentrations, if mean values were not provided in the manuscript.

| **Habitat** | **# samples**  **day** | **March*** | **May** | **July** | | **August** | | **December** | |
| --- | --- | --- | --- | --- | --- | --- | --- | --- | --- |
|  |  | Q_0.05_ - Q_0.95_ | Q_0.05_ - Q_0.95_ | Q_0.05_ - Q_0.95_ | | Q_0.05_ - Q_0.95_ | | Q_0.05_ - Q_0.95_ | |
|  |  |  |  |  |  |  |  |  |  |
| **Algae-dominated** | **1** | 50 - 101 | 19 - 66 | 56 - 137 | | 49 - 225 | | 9 - 17 | |
|  | **5** | 55 - 87 | 25 - 52 | 65 - 100 | | 94 - 196 | | 10 - 16 | |
|  | **10** | 59 - 80 | 30 - 47 | 66 - 95 | | 101 - 186 | | 11 - 15 | |
|  | **25** | 60 - 78 | 34 - 45 | 69 - 81 | | 129 - 173 | | 11 - 14 | |
|  | **50** | 63 - 72 | 36 - 44 | 69 - 77 | | 135 - 165 | | 11 - 13 | |
| **Mixed-vegetated** | **1** | 60 - 110 | 22 - 80 | 69 - 180 | | 90 - 320 | | 7 - 31 | |
|  | **5** | 64 - 106 | 36 - 69 | 96 - 155 | | 145 - 255 | | 11 - 25 | |
|  | **10** | 71 - 101 | 40 - 67 | 102 - 138 | | 158 - 235 | | 13 - 23 | |
|  | **25** | 75 - 95 | 44 - 64 | 105 - 126 | | 164 - 215 | | 14 - 22 | |
|  | **50** | 80 - 89 | 48 - 63 | 107 - 123 | | 171 - 209 | | 16 - 21 | |
| **Bare sediments** | **1** | 49 - 103 | 27 - 52 | 45 - 96 | | 62 - 253 | | 7 - 14 | |
|  | **5** | 53 - 96 | 34 - 46 | 53 - 83 | | 117 - 214 | | 7 - 11 | |
|  | **10** | 60 - 86 | 36 - 45 | 55 - 82 | | 132 - 187 | | 8 - 10 | |
|  | **25** | 66 - 81 | 39 - 43 | 59 - 81 | | 137 - 174 | | 8 - 9 | |
|  | **50** | 70 - 77 | 40 - 43 | 65 - 80 | | 140 - 167 | | 8 - 9 | |

Table S6. Results of bootstrapping the population of CH_4_ concentrations observed, with a sampling number ranging from 1 to 100 samples per day, and 200 replicates for each number of samples. Results presented here show the 5th – 95th percentiles (Q_0.05_ – Q_0.95_) of the CH_4_ concentration for 1, 5, 10, 25, and 50 discrete samples per day. The asterisk denotes under-ice sampling in March.

**SI References**

1. O. Schmale, *et al.*, Distribution of methane in the water column of the Baltic Sea. *Geophys. Res. Lett.* **37**, 1–5 (2010).

2. S. T. Wilson, *et al.*, An intercomparison of oceanic methane and nitrous oxide measurements. *Biogeosciences* **15**, 5891–5907 (2018).

3. W. Gülzow, G. Rehder, J. Schneider v. Deimling, T. Seifert, Z. Tóth, One year of continuous measurements constraining methane emissions from the Baltic Sea to the atmosphere using a ship of opportunity. *Biogeosciences* **10**, 81–99 (2013).

4. A. N. Dzyuban, I. N. Krylova, I. A. Kuznetsova, Properties of bacteria distribution and gas regime within the water column of the Baltic Sea in winter. *Oceanology* **39**, 348–351 (1999).

5. C. Humborg, *et al.*, High Emissions of Carbon Dioxide and Methane From the Coastal Baltic Sea at the End of a Summer Heat Wave. *Front. Mar. Sci.* **6**, 1–14 (2019).

6. X. Ma, M. Sun, S. T. Lennartz, H. W. Bange, A decade of methane measurements at the Boknis Eck Time Series Station in Eckernförde Bay (southwestern Baltic Sea). *Biogeosciences* **17**, 3427–3438 (2020).

7. N.-X. Geilfus, *et al.*, Landfast sea ice in the Bothnian Bay (Baltic Sea) as a temporary storage compartment for greenhouse gases. *Elem. Sci. Anthr.* **9** (2021).

8. M. Lundevall-Zara, E. Lundevall-Zara, V. Brüchert, Sea-Air Exchange of Methane in Shallow Inshore Areas of the Baltic Sea. *Front. Mar. Sci.* **8**, 1–20 (2021).

9. A. V. Borges, W. Champenois, N. Gypens, B. Delille, J. Harlay, Massive marine methane emissions from near-shore shallow coastal areas. *Sci. Rep.* **6**, 2–9 (2016).

10. T. Weber, N. A. Wiseman, A. Kock, Global ocean methane emissions dominated by shallow coastal waters. *Nat. Commun.* **10**, 1–10 (2019).
